# Elastic Properties of Thermoplastic Polyurethane Fabricated Using Multi Jet Fusion Additive Technology

**DOI:** 10.3390/polym17101363

**Published:** 2025-05-16

**Authors:** Karolina Wilińska, Marta Kozuń, Celina Pezowicz

**Affiliations:** Department of Mechanics, Materials and Biomedical Engineering, Wrocław University of Science and Technology, 50-370 Wrocław, Polandcelina.pezowicz@pwr.edu.pl (C.P.)

**Keywords:** additive manufacturing, thermoplastic polyurethane, elastomers

## Abstract

This study investigates the elastic properties of thermoplastic polyurethane (TPU) produced through Multi Jet Fusion (MJF) (HP Inc., Palo Alto, CA, USA) additive technology. TPU specimens of varying thicknesses (0.5 mm to 1.0 mm) and orientations (horizontal, diagonal, vertical) were tested. Results show anisotropic behavior, with diagonally oriented specimens exhibiting the highest elastic properties. The study emphasizes the importance of specifying the method for determining elastic properties in TPU filaments for accurate material selection in applications. The findings highlight that a single-value Young’s modulus is insufficient to describe TPU’s elastic behavior, emphasizing the need for more detailed methodological specification in material datasheets. Additionally, SEM (Thermo Fisher Scientific, Waltham, MA, USA). analysis reveals that build orientation significantly affects failure modes in MJF-printed TPU: vertical prints tend to fracture in a brittle-like manner due to interlayer delamination, whereas horizontal and diagonal orientations promote ductile failure with better layer cohesion. These insights are critical for both accurate material selection and for optimizing TPU parts in functional applications, particularly where mechanical performance under tension is essential.

## 1. Introduction

Thermoplastic polyurethanes (TPUs) are copolymers with hard and soft segments forming a two-phase microstructure [1], and they belong to the group of elastomers [2]. The composition and structure of these segments significantly influence the mechanical properties of the material in terms of tensile strength and elongation [3]. TPU properties can be modified by controlling the ratio of soft and hard segments and structural morphologies, making it possible to obtain excellent wear resistance, high tensile strength, good chemical resistance, and machinability [4,5]. Compared to other elastomeric materials, TPUs can be recycled and reused through thermoplastic processing, which significantly reduces environmental pollution [6,7,8].

Research conducted so far on the mechanical properties of polymer materials manufactured using additive technologies has shown that the values of the mechanical parameters are significantly affected by factors such as specimen thickness [9], infill line orientation [5], specimen orientation relative to the build platform of the printer [10,11,12,13], and printing parameters (e.g., layer height, printing speed) [11,14,15]. Xiao et al. [5] presented the influence of printing parameters such as infill line angle or temperature (FDM technology) on the mechanical properties of medical TPUs. The authors showed that medical TPU has a tensile strength of 46.7 MPa and break elongation of 702% (at a speed rate of 500 mm/min). According to the authors, the infill line angle of 45° and the printing temperature of 215 °C are optimal for the TPU medical printing process using FDM technology from the point of view of the mechanical properties of the produced specimens. Qi et al. [16] showed that in a uniaxial tensile test TPU exhibits very complicated stress-strain behavior, which has strong rate dependence, hysteresis, and softening, where the softening is evident upon reloading. Xu et al. [14] studied the effect of three processing parameters on the mechanical properties of 3D-printed TPU parts, including build orientation, post-processing, and mix ratio. The authors showed that the mix ratio of the new powder has the greatest impact on the mechanical properties of TPU specimens obtained using SLS technology. The new powder has better particle quality and thermal properties, which are more effective for SLS processing. According to the above authors, TPU specimens printed in a flat orientation show better mechanical properties. For specimens printed in a vertical orientation, the tensile strength is 40% lower, and deformability is about 60% lower compared to a flat orientation. Tey et al. [12] showed that the built orientation of TPU specimens made using MJF technology affects their mechanical properties (i.e., tensile stress, Young’s modulus, break strain). The highest values of tensile stress (50.9 MPa) and Young’s modulus (1319.8 MPa) and the lowest value of break strain (32.1%) are found in specimens oriented vertically relative to the build platform of the printer.

In recent years, the incorporation of nanomaterials—especially carbon nanotubes (CNTs) and graphene—into thermoplastic polyurethane (TPU) matrices has attracted significant attention due to their ability to enhance mechanical, thermal, and electrical properties. Shchegolkov et al., 2025 [17] provide a comprehensive review of CNT-based nanocomposites, showing that CNTs improve polymer strain sensitivity, enable temperature self-regulation in heating applications, and enhance thermal and electrical conductivity even at low concentrations, which helps preserve the elastomeric properties of TPU. These functionalized polymer nanocomposites (FPNs) can exhibit effects such as shape memory, self-healing, and dynamic response to environmental stimuli, qualifying them as “smart materials”. Moreover, the combination of CNTs with other nanofillers such as graphene and nano-aluminum allows for synergistic enhancements, including improved load distribution and conductivity networks. Morphological studies confirm that combining multi-walled CNTs (MWCNTs) with graphene nanoplatelets (GNPs) in TPU results in complementary conductive pathways that optimize both electrical and mechanical performance. For instance, TPU composites with a 1:2 MWCNT-GNP ratio show well-dispersed, dense conductive networks, making them suitable for strain sensors and wearable electronics.

Other researchers corroborate these findings. Liu et al., 2016 [18] demonstrated that CNT-graphene hybrid TPU composites exhibit superior strain-sensing capability and electrical conductivity due to synergistic filler interactions. Similarly, Roy et al., 2015 [19] reported improved thermal stability and stiffness in TPU nanocomposites incorporating MWCNTs and reduced graphene oxide. These enhancements are achieved through better filler dispersion and interfacial bonding.

In the studies presented above, the most frequently determined mechanical parameters for TPUs and other elastomeric materials are tensile/compressive strength and strain/elongation at break. The presented research also showed that the above-mentioned mechanical properties are affected by such parameters as specimen thickness, orientation relative to the build platform, printing parameters, and infill pattern. The determination of Young’s modulus of elasticity is a key aspect of material characterization, allowing assessment of the stiffness and deformation behavior of a material under different conditions [20]. In studies where this issue is addressed, elasticity is most often determined by providing the value referred to as Young’s modulus [12] or the stiffness parameter expressed in pascals [21]. According to Bednarz [22], in the case of elastomeric materials whose relationship between strain and stress is not linear, the determination of elasticity by giving a single value described as Young’s modulus is imprecise and requires the use of a method other than the classic one. Therefore, the aim of this study is to characterize the elastic properties of thermoplastic polyurethane produced using the Multi Jet Fusion (MJF) additive technology from HP. The tests used different specimen thicknesses (0.5–1.0 mm) and different build orientations (vertical, horizontal, diagonal).

## 2. Materials and Methods

**Figure 1:**
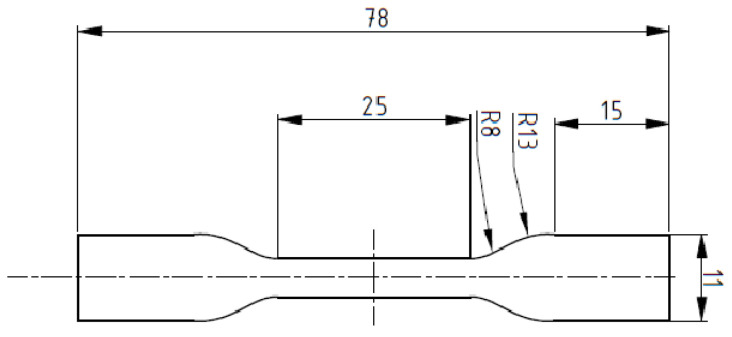
Dimensions of the produced specimen ASTM D638-10 and ASTM D4482-11 standards.

The research material was Ultrasint TPU from BASF. Dumbbell specimens were produced to test the mechanical properties (compliance with ASTM D638-10 [23] and ASTM D4482-11 standards [24]). The specimen dimensions are shown in Figure 1.

The specimens were produced in cooperation with 3D Center sp. z o.o. in Wroclaw. The thicknesses of the specimens were 0.5 mm, 0.6 mm, 0.7 mm, 0.8 mm, 0.9 mm, and 1.0 mm. The specimens were produced with MJF additive technology using an HP Jet Fusion 5210 3D printer (HP Inc., Palo Alto, CA, USA). The ratio of new powder to used powder was 20:80.

**Figure 2:**
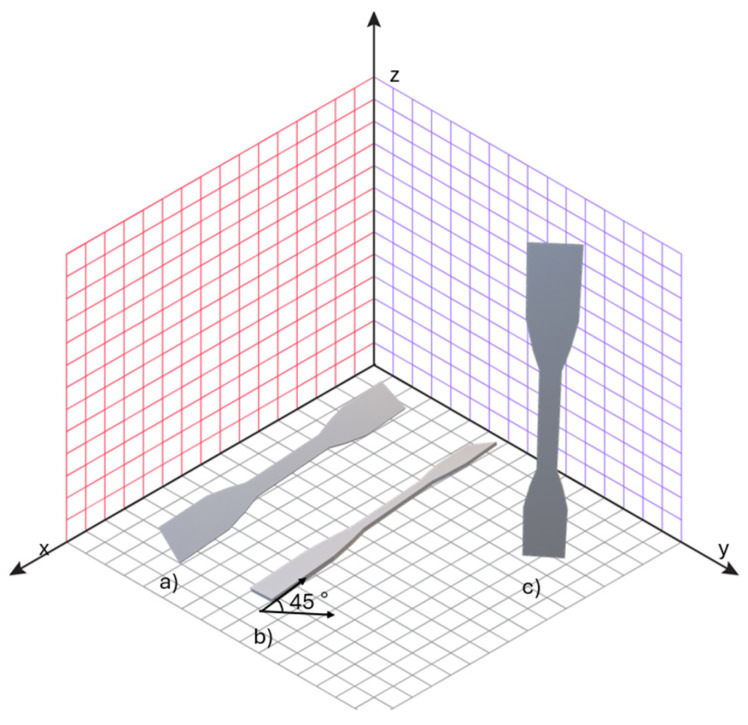
Arrangement of samples in the working chamber: (**a**) horizontal; (**b**) diagonal; (**c**) vertical.

The specimens were produced in different orientations relative to the printer’s build platform on which the print is made, as shown in Figure 2. After printing, the specimens were mechanically processed, i.e., sandblasted (DyeMansion sandblaster (DyeMansion GmbH, Planegg, Germany)) using 300–400 μm diameter glass beads to remove adherent powder particles. Surface roughness was subsequently verified using a contact profilometer (Leica Microsystems, Wetzlar, Germany), and the average roughness (Ra) ranged from 20 to 24 µm across all specimens. The variation was minimal and consistent between groups, indicating that surface roughness did not significantly contribute to differences in mechanical properties.

**Figure 3:**
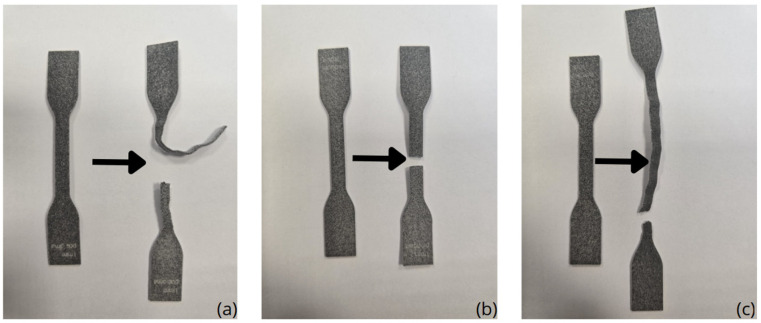
Fracture mechanism for orientation: (**a**) horizontal; (**b**) vertical; (**c**) diagonal.

The mechanical property tests included a uniaxial tensile test under static loading conditions, which was performed at a loading rate of 100 mm/min. The tests were carried out using an MTS Criterion Model 41 testing (MTS Systems Corporation, Eden Prairie, MN, USA) machine at an ambient temperature of 23 °C. During the tests, the force and displacement values of the specimen were recorded, based on which the stress-strain curves were determined. The obtained curves were used to determine the static longitudinal modulus of elasticity, i.e., segmental secant modulus and tangent modulus. The specimens were subjected to tensile testing until failure, and the fracture mechanism for each build orientation (vertical, horizontal, and diagonal) is presented in Figure 3.

The samples were tensile tested to failure, and the failure mechanism of the vertical, horizontal, and diagonal build orientations is displayed in Figure 3. It can be observed that the fracture patterns of the diagonal and horizontal orientations exhibit the same failure mode, which is a wavy, accordion-like deformation typical of elastomeric materials under tensile stress. In contrast, the vertical specimens exhibit a distinctly different fracture mechanism, with a clean, brittle-like fracture, most likely along the layers that were printed. The vertical specimens also exhibited the lowest tensile strength of all the orientations, again demonstrating the influence of build direction on mechanical performance.

**Figure 4:**
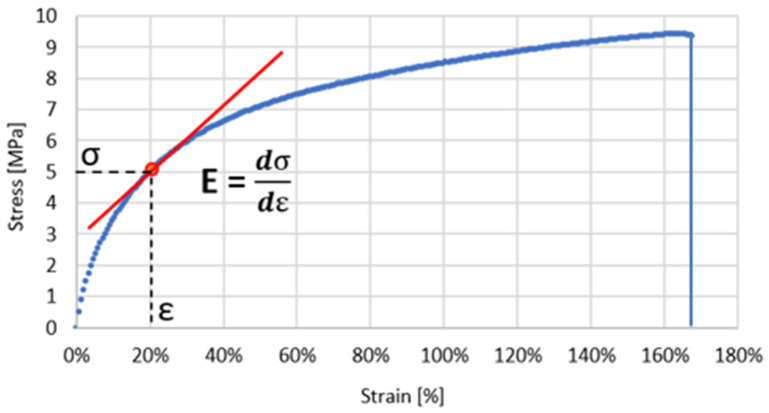
Methods of determining the longitudinal modulus of elasticity for chord modulus. The blue dotted line represents the experimental stress-strain curve. The red line denotes the linear approximation used to calculate the chord modulus between two selected points on the curve.

**Figure 5:**
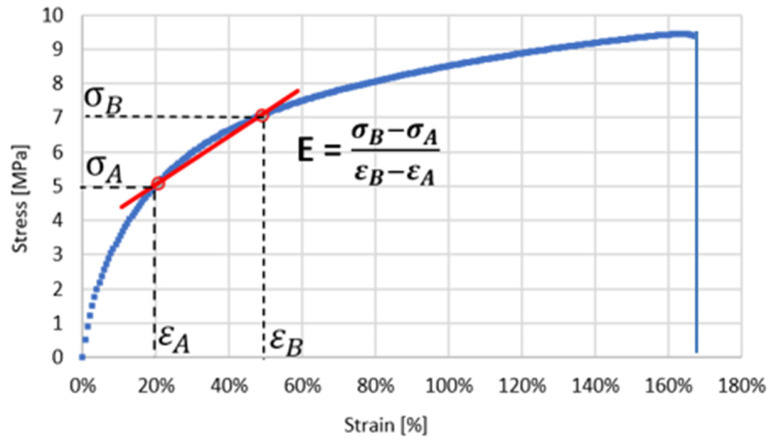
Methods of determining the longitudinal modulus of elasticity for tangent modulus. The blue dotted line represents the experimental stress-strain curve. The red line is the tangent to the curve at the selected point, used to calculate the tangent modulus.

The sectional secant modulus was determined as the slope of the curve between two given points on the stress-strain curve. The following ranges of strain were adopted (εrel.): from 10 to 20% (E10–20%), from 20 to 50% (E20–50%), from 50 to 70% (E50–70%), and from 70 to 100% (E70–100%). Additionally, to assess the degree of non-linearity of the material, the non-linearity index was determined, which was defined as the ratio of the secant modulus obtained for the range of 70–100% (E70–100%) to the secant modulus obtained for the initial section, i.e., in the range of 10–20% (E10–20%). This parameter was determined as follows: E_(70–100%)/E_(10–20%). The tangent modulus was determined as the slope of the stress-strain curve for the strain values of 10% (E10%), 20% (E20%), 50% (E50%), 70% (E70%), and 100% (E100%). The method of determining the elastic moduli is shown in Figure 4 and Figure 5.

**Figure 6:**
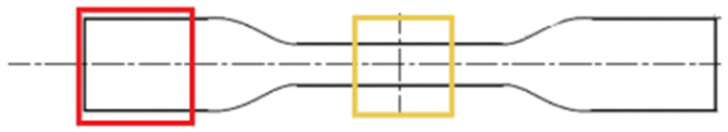
Schematic representation of the tensile specimen indicating locations of SEM observations. The red square marks the region examined before testing (unloaded state), while the yellow square indicates the fracture surface analyzed after tensile failure.

To investigate the surface morphology and failure characteristics of the TPU specimens before and after mechanical testing, scanning electron microscopy (SEM) was conducted. The observations were performed using a Phenom ProX SEM system operated at an accelerating voltage of 10 kV in backscattered electron mode. Samples were analyzed without conductive coating, as the TPU material provided sufficient contrast under the given imaging conditions. Representative SEM micrographs revealed the characteristic layered structure and fracture features of the MJF-printed TPU. A schematic illustration indicating the location of the SEM analysis, performed on the fracture surface of the tensile specimen, is shown in Figure 6.

An X-ray diffraction (XRD) analysis was conducted to examine the crystallographic structure of the TPU samples. Measurements were performed using a step-scan mode in the 2θ range of 5–90° to initially identify the relevant diffraction angles. A refined scan was then carried out in a narrowed 2θ range of 3–60°, followed by a high-resolution scan within the same range to improve peak accuracy. The resulting diffraction patterns were used to assess the degree of crystallinity and identify the characteristic diffraction peaks of the material.

### Statistical Analysis

The statistical analysis was conducted using Prism 7 (GraphPad Software, San Diego, CA, USA) and Statistica 13.3 (TIBCO) software. Firstly, Grubbs’ test was used to identify the outliers and then the normal distribution of each analyzed parameter was verified using the Shapiro-Wilk normality test (α = 0.05). The statistical significance of the differences between the groups was tested using the Friedman test and the one-way repeated measures ANOVA with Tukey’s post hoc test. All stages of statistical analysis were performed at a significance level of *p* < 0.05. The values of the mechanical parameters are presented as the median.

## 3. Results

**Figure 7:**
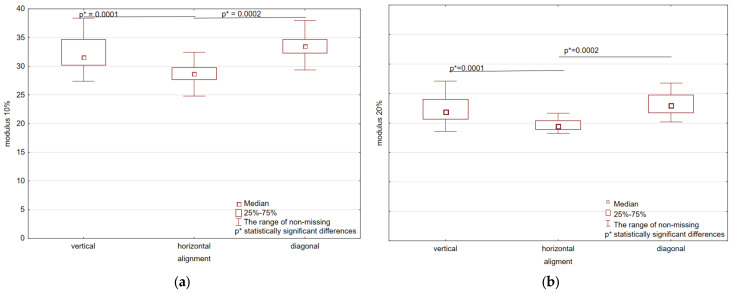

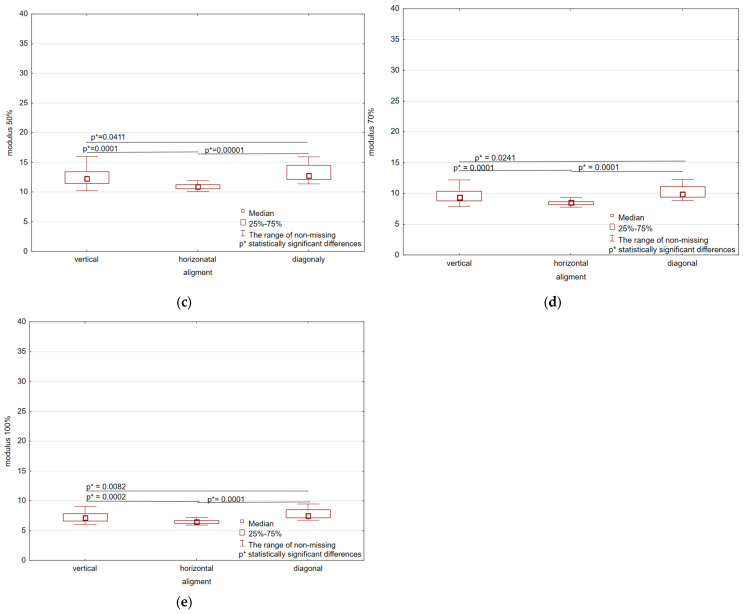
Statistical analysis with statistically significant differences for tangent modulus: (**a**) modulus 10%; (**b**) modulus 20%; (**c**) modulus 50%; (**d**) modulus 70%; (**e**) modulus 100%.

**Figure 8:**
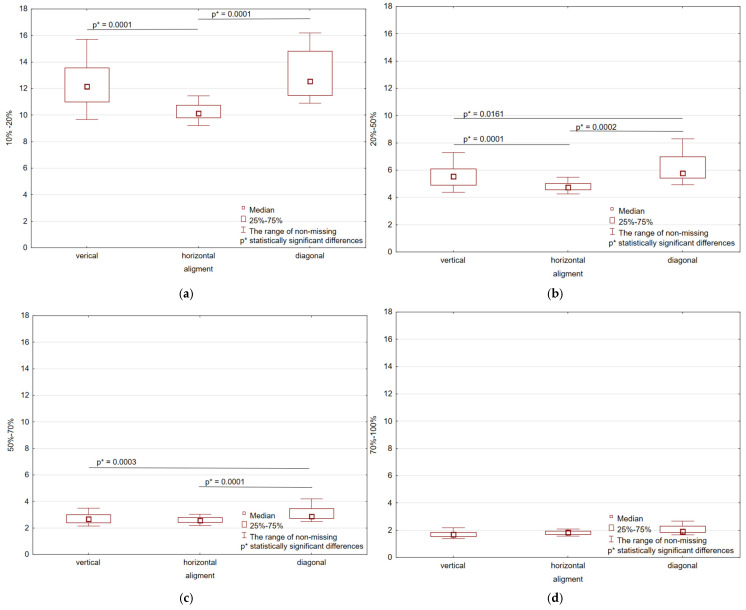
Statistical analysis with statistically significant differences for chord modulus: (**a**) 10–20%; (**b**) 20–50%; (**c**) 50–70%; (**d**) 70–100%.

The conducted tests allowed us to obtain stress-strain curves (σ–ε). All of the resulting curves are characterized by non-linearity. Similar curves for TPU produced using additive technology were also obtained by Xu et al. [14]. Due to the above-mentioned non-linearity, tangent modulus of elasticity and sectional chord modulus were determined as the measure of elasticity of the tested material. Results of the statistical analysis are shown in Figure 7 and Figure 8.

The method for determining statistically significant differences was included in the methodology section. The analysis was conducted based on the arrangement in the working chamber (Figure 2) as well as thickness. For all strain levels, the horizontal alignment consistently exhibits the smallest tangent modulus values, which indicates lesser stiffness for this orientation. The vertical and diagonal orientations, however, exhibit considerably greater modulus values, indicating superior mechanical performance. These differences are even more pronounced at lower levels of strain (10–50%), where both vertical and diagonal orientations exhibit very much larger modulus values compared to that of horizontal orientation. With rising strain level, the overall modulus values decrease, as anticipated with the material softening with greater deformation. At 100% strain (Figure 7e), the modulus values are lowest for all orientations, with the weakest still being the horizontal alignment. Statistically significant differences (*p* < 0.05) are observed between horizontal vs. vertical and horizontal vs. diagonal orientations, demonstrating that infill alignment has a significant influence on mechanical properties even at peak strain. Median tangent modulus values are shown in Table 1.

At the 10–20% strain level (Figure 8a), the highest modulus values are observed in the diagonal and vertical directions, with the horizontal alignment possessing considerably lower stiffness. Statistically significant differences (*p* < 0.05) are observed between the horizontal direction and the vertical and diagonal alignments. When strain is increased to the 20–50% (Figure 8b), overall modulus values decrease, but the trend remains the same: horizontal alignment remains lowest in stiffness, and diagonal and vertical orientations remain significantly higher. Statistical analysis reveals significant differences (*p* < 0.05) for all orientation pairs, revealing a strong infill direction effect on stiffness within this range of strain. At the 50–70% strain (Figure 8c), additional decreases in modulus values are observed, replicating the expected material behavior to progressive deformation. The horizontal orientation remains the least stiff; however, significant differences were observed between horizontal and diagonal group and vertical and diagonal group. In the 70–100% strain (Figure 8d), minimum modulus values are observed across all orientations, supporting progressive stiffness reduction at high strain ranges. These findings demonstrate the significant effect of infill direction on mechanical behavior, with horizontal orientation producing the lowest tangent modulus values throughout, whereas vertical and diagonal directions maintain greater stiffness across all ranges of strains. The statistical significance shown here reveals that the selection of infill direction is extremely critical for structural optimization, particularly for applications requiring mechanical resilience to progressive loading, such as 3D-printed goods and composite parts. Median values of chord modulus for different orientations are shown in Table 2.

The values of the tangent modulus and chord modulus are shown as a median in Table 3 and Table 4.

As shown in the tables, the highest values of modulus, for both calculation methods, were generally observed in specimens with a thickness of 0.6 mm. In contrast, the lowest values were generally recorded for the thinnest (0.5 mm) or thickest (1.0 mm) specimens, depending on the type of modulus and strain range. A marked decrease in modulus values is observed with increasing strain, reflecting the expected softening behavior of elastomeric materials at higher strain.

**Figure 9:**
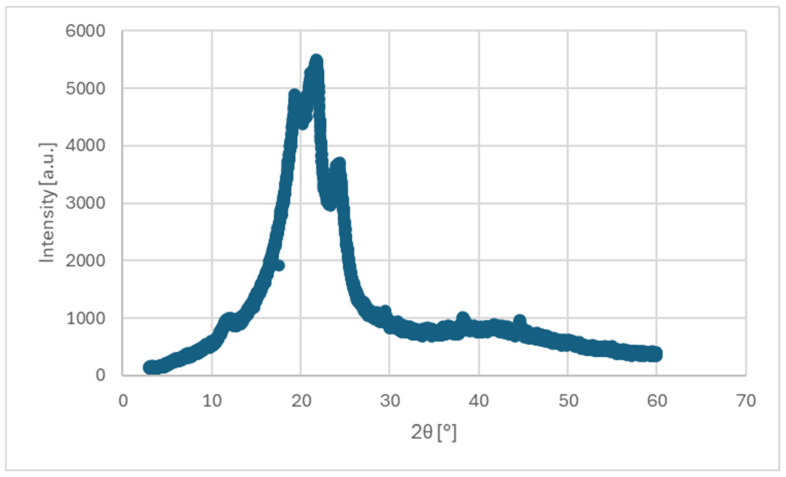
High-resolution XRD pattern of TPU sample showing broad peaks around 19.2° and 22.3° 2θ, indicating low crystallinity and short-range order typical of segmented polyurethane.

The XRD pattern of the TPU sample (shown in Figure 9) exhibits two broad diffraction peaks centered around 19.2° and 22.3° 2θ, which are characteristic of semi-crystalline thermoplastic polyurethane [25]. These peaks correspond to the ordered arrangement of hard segment domains embedded in an amorphous soft matrix. The broad shape of the peaks indicates a low degree of crystallinity and the presence of short-range order, typical for segmented polyurethanes. No sharp crystalline peaks were observed, confirming the predominantly amorphous nature of the material with only partial microphase crystallization.

**Figure 10:**
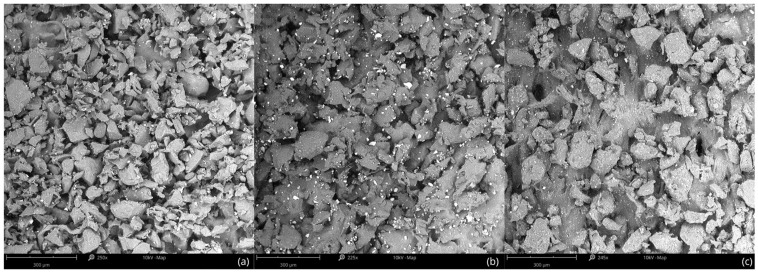
SEM images of the TPU surface structure before mechanical testing for different sample orientations: (**a**) vertical; (**b**) horizontal; (**c**) diagonal.

The microstructure of the TPU specimen before mechanical testing is shown in Figure 10.

As shown in Figure 10, the surface morphology of TPU samples before mechanical tests appears consistent across all build orientations (vertical, horizontal, and diagonal). SEM images reveal a granular, irregular texture typical for MJF-printed elastomers, with no significant differences in particle distribution or surface roughness that could explain the variation in elastic properties. This indicates that the observed anisotropy in mechanical performance is not due to initial microstructural differences, but rather to the internal architecture and layer alignment introduced during the additive manufacturing process. The microstructure of the TPU specimen after mechanical testing is shown in Figure 9.

**Figure 11:**
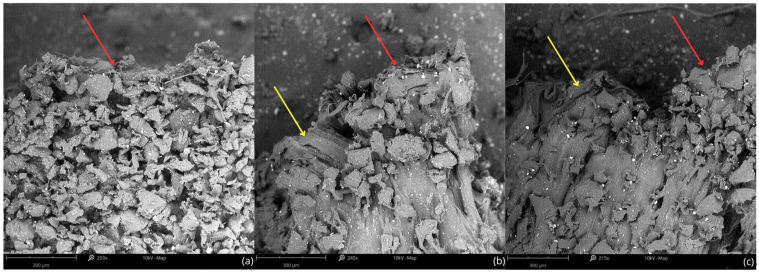
SEM images of TPU fracture surfaces after tensile testing for different build orientations: (**a**) vertical; (**b**) horizontal; (**c**) diagonal. Red arrows indicate brittle-like features associated with interlayer delamination, particularly visible in vertically built specimens. Yellow arrows indicate ductile tearing patterns with stretched fibrillar structures, visible in horizontal and diagonal samples (Figure 2b,c).

Fractographic observations using SEM (Figure 11) revealed differences in the failure mechanisms depending on the build orientation of the specimens. In vertically printed samples (Figure 11a), the fracture surface appeared more irregular and fragmented, indicating a brittle-like fracture mechanism. This suggests that the crack propagated along interlayer boundaries, most likely due to weaker adhesion between layers when the load was applied perpendicular to the build direction. This observation aligns with the mechanical test results, where vertically oriented specimens exhibited lower tensile strength and reduced deformability compared to other orientations.

In contrast, the fracture surfaces of horizontally (Figure 11b) and diagonally (Figure 11c) printed specimens showed typical features of ductile failure, including layered, fibrillar deformation and rough tearing patterns (see yellow arrows in Figure 11b,c). These features indicate greater energy absorption and plastic deformation prior to failure, consistent with the higher values of tangent and chord modulus obtained for these orientations, particularly the diagonal. The similarity between the horizontal and diagonal fracture morphologies suggests a comparable deformation mechanism, although diagonal samples achieved the most favorable elastic response overall.

## 4. Discussion

This study investigates the influence of build orientation on elastic material properties of TPU specimens manufactured by MJF additive technology. The uniaxial tension tests showed non-linear stress-strain behaviors, which necessitated the use of tangent modulus and chord modulus to establish the elastic response of the material. The results confirm that build orientation significantly affects elasticity, with statistically significant differences between the different orientations.

For all tested thicknesses, the highest elastic properties were observed in diagonal orientation as both tangent and chord modulus values. For horizontally oriented specimens, the lowest modulus values were always observed, which is related to lower stiffness. This suggests that mechanical performance is enhanced in diagonal orientation, perhaps due to more effective load distribution across the structure, as well as the fact that the horizontal orientation is mechanically weaker when tensile stresses are applied.

The findings concur with other research, particularly the work by Tey et al., 2021 [12], which found that vertically printed MJF-printed TPU specimens possess superior mechanical properties. Inconsistencies arise when comparing the study to other additive manufacturing methods. For instance, Xu et al., 2020 [14] reported that horizontally printed TPU samples with SLS technology were more tensile in strength and flexible than vertically printed ones. Similarly, Markiz et al., 2020 [26] reported that vertically printed ABS samples exhibited a 44.7% increase in mechanical strength compared to horizontally printed samples.

MJF, SLS, and FDM printing processes may differ, and differences between these might contribute to such discrepancies. Mechanical properties in FDM printing are not only a function of build orientation but also of the internal infill pattern and inter-layer bonding strength. Surface tension and powder sintering conditions introduce variables on mechanical behavior for SLS printing. The MJF process, being a controlled thermally fused powder deposition, has the possibility of having internally different microstructures compared to other processes [27,28].

Of particular note is that the previous work presented Young’s modulus as a value, but not as how it was determined. Because elastomers are so non-linear, a single value of Young’s modulus is insufficient to characterize material response at different levels of strain. Present work demonstrates that elastic behavior varies significantly depending on the modulus calculation method (tangent vs. chord modulus) and strain range; thus, it is essential to identify the method applied in material characterization. Comparable observations were reported by Bednarz, 2016 [22], underlining that traditional methods of measuring elasticity might not be sufficient in elastomeric materials.

TPU specimen thickness also influenced elastic response. Maximum tangent modulus was recorded for specimens of 0.6 mm, while the minimum was observed for 0.5 mm specimens. Similarly, for chord modulus, the maximum was for 0.6 mm specimens, while the minimum was for 1.0 mm specimens. This reading is indicative of minimizing mechanical deformation when loaded in 0.6 mm specimens, making them more resistant to strain. In contrast, lower (0.5 mm) and higher (1.0 mm) specimens recorded greater deformation, suggesting an optimum thickness in order to obtain the maximum stiffness of MJF-printed TPU.

Perhaps the most pertinent engineering implication from this study is that elastic property testing methods must be elaborated upon in the datasheets for the materials. Most producers of these materials indicate Young’s modulus without indicating that it is measured from tangent, secant, or chord modulus, and the variations can produce differences in choosing materials for use in certain applications. This paper proposes that listing more than one elasticity value, particularly at more than one level of strain, provides a fuller mechanical description that facilitates more confident material selection for engineering applications using TPU parts.

SEM analysis of the fracture zones (Figure 11) revealed that build orientation significantly influences the failure mechanism of MJF-printed TPU specimens. Vertically printed samples exhibited a porous and irregular fracture surface, indicative of brittle-like behavior likely caused by delamination between layers. This confirms that the weakest points under tensile loading are the interlayer interfaces when the stress is applied perpendicular to the build direction.

In contrast, horizontally and diagonally oriented specimens showed more ductile fracture morphology, with visible signs of plastic deformation and layered tearing. These patterns reflect stronger cohesion along the layer paths and correlate with the higher elastic moduli observed in mechanical tests, particularly for the diagonal orientation.

These findings highlight that build orientation not only affects elastic response but also determines the dominant failure mode. From an application perspective, diagonal and horizontal orientations are more suitable for components subjected to tensile loads, while vertical builds may require reinforcement or design adaptation to mitigate interlayer weakness.

XRD analysis confirmed the semi-crystalline nature of the TPU material, with broad diffraction peaks indicating short-range order typical for segmented polyurethane. This structural feature aligns with the observed non-linear elastic behavior.

## 5. Conclusions

This study confirms that TPU samples manufactured by MJF additive technology exhibit anisotropic mechanical behavior, with the highest elastic properties in diagonally printed samples. Under static mechanical loads, diagonally printed samples deformed significantly less than horizontally printed samples, confirming the influence of build direction on material performance. Additionally, the elastic response of TPU was found to be thickness-dependent for the specimens, with 0.6 mm specimens proving to be stiffest under all test conditions. This finding suggests that mechanical performance can be engineered by selecting an optimal thickness for TPU components produced via MJF printing. The other crucial outcome of this research is the sensitivity of elasticity to the modulus calculation method. The findings confirm that Young’s modulus cannot be a true measure of the elastic behavior of TPU, given that it has non-linear stress-strain characteristics. The outcome emphasizes the significance of the modulus calculation method to be explicitly mentioned on material datasheets by manufacturers so that there are no errors in comparison and selection of appropriate materials for different engineering applications. In summary, the research underscores the importance of build orientation, specimen thickness, and modulus measurement approach in determining the mechanical properties of TPU processed through MJF technology. Additional insights gained from SEM analysis confirmed that the mechanical behavior of MJF-printed TPU is strongly linked to the failure mechanism governed by build orientation. Brittle-like fracture features in vertically printed samples indicate interlayer delamination as the dominant failure mode, while ductile tearing in diagonal and horizontal orientations reflects better stress accommodation and layer cohesion. These observations emphasize the importance of build orientation not only in tuning elastic properties but also in ensuring structural integrity. For engineering applications requiring tensile strength and durability, diagonal and horizontal print orientations are preferable, whereas vertical orientation may require design compensation or additional reinforcement to prevent premature failure.

The findings of this study have direct practical implications for the design and optimization of components made from thermoplastic polyurethane using Multi Jet Fusion (MJF) additive manufacturing. The demonstrated anisotropic behavior—particularly the superior stiffness observed in diagonal build orientations—and the identification of optimal specimen thickness (0.6 mm) provide valuable guidance for engineers seeking to tailor mechanical performance based on structural requirements. For example, in applications such as custom orthopedic devices, flexible mechanical couplings, or protective housings, the strategic selection of build orientation and thickness can significantly enhance load-bearing capacity and durability. Moreover, the results underscore the importance of explicitly specifying the method of modulus calculation, especially for non-linear materials like TPU, in order to ensure reliable material selection and performance prediction. This is particularly relevant in industries where high strain tolerance and elastic recovery are critical, such as in biomedical, automotive, and wearable electronics sectors.

## Figures and Tables

**Table 1:** The median values of the tangent modulus expressed in MPa for specimens with different orientations determined for the following strain ranges: 10%, 20%, 50%, 70%, and 100%.

The Strain Range	Vertical	Horizontal	Diagonal
Modulus 10%	31.6	28.7	33.5
Modulus 20%	21.8	19.4	23.0
Modulus 50%	12.3	10.9	12.8
Modulus 70%	9.4	8.5	9.9
Modulus 100%	7.2	6.5	7.5

**Table 2:** The median values of the chord modulus expressed in MPa for specimens with different orientations: from 10 to 20%, from 20 to 50%, from 50 to 70%, and from 70 to 100%.

The Strain Range	Vertical	Horizontal	Diagonal
10–20%	12.2	10.2	12.5
20–50%	5.5	4.8	5.7
50–70%	2.7	2.5	2.9
70–100%	1.7	1.8	1.9

**Table 3:** The median values of the tangent modulus expressed in MPa for specimens with different thickness determined for the following strain ranges: 10%, 20%, 50%, 70%, and 100%.

The Strain Range	0.5 mm	0.6 mm	0.7 mm	0.8 mm	0.9 mm	1 mm
Modulus 10%	30.6	32.5	29.9	31.7	31.8	31.1
Modulus 20%	21.9	22.9	20.5	21.6	21.7	21.2
Modulus 50%	12.4	12.9	11.4	11.9	12.0	11.7
Modulus 70%	9.6	10.0	8.8	9.3	9.3	9.1
Modulus 100%	7.3	7.6	6.7	7.1	7.1	6.9

**Table 4:** The median values of the chord modulus expressed in MPa for specimens with different thickness: from 10 to 20%, from 20 to 50%, from 50 to 70%, and from 70 to 100%.

The Strain Range	0.5 mm	0.6 mm	0.7 mm	0.8 mm	0.9 mm	1 mm
10–20%	13.5	13.1	11.0	11.1	11.4	11.0
20–50%	5.8	5.9	5.0	5.2	5.4	5.1
50–70%	2.8	2.9	2.6	2.7	2.8	2.7
70–100%	1.9	1.9	1.8	1.8	1.9	1.7

## Data Availability

The data presented in this study are available on request from the corresponding author. The data are not publicly available due to institutional restrictions.
